# Fibrosis in the kidney: is a problem shared a problem halved?

**DOI:** 10.1186/1755-1536-5-S1-S14

**Published:** 2012-06-06

**Authors:** Tim D Hewitson

**Affiliations:** 1 Department of Nephrology, Royal Melbourne Hospital & Department of Medicine, University of Melbourne, Melbourne, Australia

## Abstract

Fibrotic disorders are commonplace, take many forms and can be life-threatening. No better example of this exists than the progressive fibrosis that accompanies all chronic renal disease. Renal fibrosis is a direct consequence of the kidney's limited capacity to regenerate after injury. Renal scarring results in a progressive loss of renal function, ultimately leading to end-stage renal failure and a requirement for dialysis or kidney transplantation.

Although it manifests itself histologically as an increase in extracellular matrix, we know that the histological appearance can be caused by a de novo synthesis of matrix (primarily collagen), or a disproportionate loss of renal parenchyma. In both cases the process depends on a resident mesenchymal cell, the so-called myofibroblast, and is independent of disease etiology. Potentially we can ameliorate fibrosis, either indirectly by modifying the environment the kidney functions in, or more directly by interfering with activation and function of myofibroblasts.

However, while renal fibrosis shares many features in common with the wound healing response in other organs, we also recognise that the consequences can be highly kidney specific. This review highlights the similarities and differences between this process in the kidney and other organs, and considers the therapeutic implications.

## Introduction

Fibrosis involves an excess accumulation of extracellular matrix (primarily composed of collagen) and usually results in loss of function when normal tissue is replaced with scar tissue [1]. No better example of this exists than the progressive fibrosis that accompanies all chronic renal disease. However, an overview of renal disease suggests that complementary but different mechanisms are responsible for fibrosis. Likewise, although there are obvious parallels between fibrosis in the kidney and elsewhere, there are also a number of important differences, and kidney specific consequences, that distinguish progressive renal disease. The purpose of this review is to summarise the mechanisms of renal fibrosis and its causes and consequences. In doing so it will emphasise the similarities and differences between the renal response and that of other organs.

## Discussion

### Etiology of renal disease

**Figure 1:**
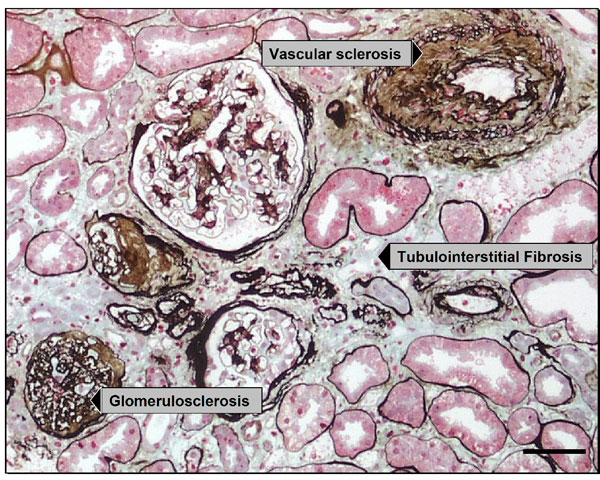
Histology of end-stage kidney disease consists of tubulointerstitial fibrosis (middle), glomerulosclerosis (bottom left) and vascular sclerosis (top right). Silver methenamine/Masson trichrome stain. Scale bar = 50 μm.

Kidney disease consists of a diverse range of etiologies, including immunological, mechanical, metabolic and toxic insults amongst others. These variously affect the three functional compartments of the kidney; the vasculature, glomerulus and tubulointerstitium. It is these compartments that are collectively responsible for the delivery of blood, plasma filtration and modification of the glomerular filtrate respectively. Regardless of etiology, all patients with chronic kidney disease show a decline in renal function with time [2]. The process is irreversible, inevitably leading to end-stage renal failure, a condition that requires life-long dialysis or renal transplantation. Histologically end-stage kidney disease manifests itself as fibrotic lesions affecting each compartment; glomerulosclerosis, vascular sclerosis and tubulointerstitial fibrosis (Figure 1). Even though matrix synthesis is of course part of the normal repair process that occurs after injury, excessive synthesis of extracellular matrix is itself destructive, further exacerbating injury in a vicious cycle.

### How does fibrosis develop?

We perhaps most commonly associate scarring with an excess synthesis of matrix, usually collagen. Keloids for instance represent a quintessential example of scarring which results from aberrant matrix synthesis. Nevertheless, although keloids are an extreme case, mechanistically similar processes occur in deep organ fibrosis. There are certainly renal parallels of this process, such as the focal scarring that accompanies a localised tissue trauma.

What does however now seem clear, is that aberrant matrix synthesis is only part of the process [3]. Temporal studies in experimental renal infection indicate that aberrant collagen synthesis is often transient, peaking in the first few days after infection. Histologically however, scarring as defined by increasing matrix density, continues to increase [4].

How can we account for this discrepancy? Although it has long been known that end-stage kidneys are smaller than their unscarred counterparts, it is the focal lesions found in diseases such as reflux nephropathy that provide us with a clue. The irregular surface of these kidneys indicates underlying scar tissue, highlighting the fibro-contractive nature of renal scarring. Once again, there are established non-renal examples of this process. Wound contraction has long been recognised as an integral part of skin wound healing, with the drawing together of wound edges an important part of wound closure [5]. Direct renal evidence comes from examining the histology in experimental renal infection and scarring. Being a primary tubulointerstitial model of injury, the glomeruli are largely unaffected during fibrosis, the density of glomeruli therefore providing a measure of parenchymal collapse. In what we term the "balloon" hypothesis [3], fibrosis is therefore due not only to an increase in matrix synthesis but also to the collapse of the renal parenchyma. Analogous to deflating a balloon, we are effectively measuring the same amount of matrix in a smaller volume. Morphometric studies in this model show that the combined effect of an acute increase in collagen expression and a later collapse of the renal parenchyma account for the progressive increase in scar tissue [4].

### What is the cellular basis of this process?

Fibrosis in all three anatomical compartments is associated with the activities of a mesenchyme derived cell. The interstitial fibroblast, glomerular mesangial cell and vascular smooth muscle cell are phenotypically similar, with the fibroblast and mesangial cell acquiring features of smooth muscle when activated [5-7].

In each compartment the resident mesenchymal cell is not only the principal extracellular matrix producing cell, but is also the force for contraction and reorganisation of extracellular matrix, thereby increasing its density [5].

The renal interstitial fibroblast is typical of all three scenarios and has been the focus of our studies of tubulointerstitial fibrosis. Recognised by its de novo synthesis of αsmooth muscle actin (αSMA), activated fibroblasts, so-called myofibroblasts, are a feature of all forms of progressive renal disease where their accumulation is strongly associated with disease progression [5,7]. Myofibroblasts are probably derived from multiple sources, including not only resident fibroblasts, but also pericytes, blood borne precursors and transition of adjacent epithelial cells and endothelial cells, the relative importance of each being the subject of much conjecture [8].

Regardless, myofibroblasts proliferate locally, synthesise the extracellular matrix components that constitute interstitial fibrosis and contract and reorganise matrix to increase its density. Epigenetic modifications lead to persistent activation of the protooncogene Ras, perpetual fibroblast proliferation and fibrogenesis [9]. These changes are stable modifications that can be inherited through multiple cell divisions. In what is a vicious cycle, interstitial fibrosis is probably a cause of mechanical injury and through a reduction in vascularization, increases hypoxia [10].

### Pro-fibrotic influences

Interstitial fibroblast function and differentiation is regulated by a plethora of factors, with a variety of molecules shown to directly influence renal mesenchymal cells in vitro [5]. Differentiation of myofibroblasts, proliferation and collagen synthesis are stimulated by a variety of cytokines and growth factors derived from adjacent stimulated tubular epithelial cells, endothelial cells, leukocytes or from fibroblast themselves. Other influences include mechanical forces and extracellular matrix itself. A hierarchy exists amongst the profibrotic growth factors, with the most compelling evidence being for transforming growth factor β1 (TGFβ1) and platelet derived growth factor (PDGF). Where kidney injury is persistent or repeated, increasing numbers of injured epithelial cells stall between the G2 and M phases of the cell cycle, which results in an ongoing production of TGFβ1 [11]. It is also likely that fibroblasts are stimulated more directly in some diseases because high glucose concentrations [12] and angiotensin II [13] stimulate in vitro renal cortical fibroblast proliferation and collagen production.

Likewise the contraction of renal parenchyma is under cytokine control. Most of what we know about collagen reorganisation comes from in vitro studies of collagen gel contraction and reorganisation. In a process that is directly proportional to the number of cells present, fibroblasts embedded in solidified collagen progressively contract matrix to reduce gel diameter and increase matrix density. We know that this process is dependent upon β1 integrins found on the surface of renal fibroblasts. Blocking these receptors with specific antisera is sufficient to abrogate fibroblasts binding to collagen I, and in doing so abrogate gel contraction [14]. Again however, the process is complex and is due both to *contraction *in the surrounding matrix, in the same way as a sea anemone retracts its tentacles, and *traction *or migration of fibroblasts through surrounding matrix, akin to the movement of a spider through its web, pulling on the filaments.

### Endogenous anti-fibrotic factors

As our understanding of myofibroblast function has increased, we have come to appreciate that the actions of pro-fibrotic factors are counteracted by the activities of endogenous reno-protective agents [15]. Theoretically at least it is the balance of these opposing factors that determine progression [16]. Several have been characterised including hepatocyte growth factor [17], bone morphogenic protein-7 [18] and the hormone relaxin [19,20].

### A common pathway?

Clearly, as outlined above, renal fibrosis can result from different mechanisms - both excess matrix synthesis and contraction independently and collectively contribute. In many respects this parallels fibrosis in other organs. Kidney fibrosis, as elsewhere, is dependent upon the recruitment of a myofibroblast-like cell. The process is both fibro-proliferative, fibrogenic and fibro-contractive, and is under the influence of both pro-fibrotic factors released during injury, and endogenous reno-protective factors.

However, there are a number of differences between the kidney and other organs which impact on the in vivo consequences of injury (Table 1).

**Table 1:** Renal specific factors contributing to kidney fibrosis

	Factor	Cause
1	Complexity	Kidney consists of ~20 different cell types

2	Poor capacity for regeneration	Damaged glomeruli do not regenerate Each nephron is anatomically distinct

3	Toxins	Protein leakage and tubule uptake Uraemic milieu (>50 putative or proven toxins)
	Proteinuria	
	Uraemia	

4	Hypoxia	High tubule demand for oxygen

5	Arterial pressure dependent	Pivotal role of renin-angiotensin-aldosterone system

Firstly, and perhaps most importantly, is the inherent complexity of the kidney. The kidney consists of more than 20 different cell types. Not only does this exacerbate the in situ consequences of injury but it also affects the ability of the kidney to regenerate after an insult. The clinical reality is that the delicate structure of the glomerulus is particularly prone to damage, with very limited, if any, capacity to regenerate.

Much renal disease is associated with breakdown of the glomerular filtration barrier, and passage of excess protein. Proteinuria is toxic, excess downstream tubular reabsorption of protein results in tubular inflammation and fibrosis. Patients who develop progressive renal failure also retain solutes normally excreted by the healthy kidney. The circulating uraemic serum consists of a complex mixture of more that 50 known or putative toxins [21]; including small water bound solutes, middle molecules and protein bound molecules [22]. The influence of these toxins on renal cell function is well recognised [21], with direct evidence to show that uraemia is a permissive factor in the pathogenesis of fibrosis systemically [23].

While the kidney is well vascularised, the high oxygen consumption of tubules [24] makes them highly susceptible to any reduction in interstitial oxygen supply or delivery. It has for instance long been known that the kidney accounts for almost 25% of the body's resting oxygen consumption [25]. Oxygen deprivation is sensed through a refined molecular system whose activation has been shown to initiate renal fibrosis in animal models [10,26]. Hypoxia is also probably both a consequence and cause of further progression [10].

Finally, but not least of all, is the role of arterial pressure dependent factors. Activation of the renin-angiotensin-aldosterone system (RAAS) is pivotal to the pathogenesis of much renal disease [27], and accounts in part for the close relationship between renal disease and cardiovascular complications. Effects on fibrosis may be both indirect (hypertension and mechanical injury) and direct (angiotensin II mediated fibrogenesis and contraction).

### Therapeutic implications

Organ specific differences have therapeutic implications. Renal specific factors result in renal specific treatment strategies. For instance control of blood pressure and angiotensin II are the most proven ways of preventing progression [28] and secondary fibrosis in the kidney [29]. They however have limited application outside the reno-cardio vascular system, where hypotension prevails. Likewise protein restriction can ameliorate the toxic effects of proteinuria [30]. The challenge is therefore to find those generic anti-fibrotic strategies that have potential to ameliorate progression in multiple organs, that is, the overlap. Inhibition of the profibrotic factors, use of endogenous renoprotective factors and deletion of fibroblasts are all promising targets.

## Conclusion

In conclusion, many chronic diseases progress by fibrosis, amelioration of which can play an important role in patient management. Clinical nephrology in particular is inexorably linked to the histology of the renal biopsy . As we learn more about the histology and mechanisms of fibrosis, studies in both the kidney and elsewhere will continue to identify potential organ specific and generic therapeutic targets.

## Competing interests

The author declares that they have no competing interests.
